# Research on the Development of a Way to Modify Asphalt Mixtures with PET Recyclates

**DOI:** 10.3390/ma16186258

**Published:** 2023-09-18

**Authors:** Tomasz M. Majka, Krzysztof Adam Ostrowski, Marcin Piechaczek

**Affiliations:** 1 Department of Chemistry and Technology of Polymers, Faculty of Chemical Engineering and Technology, Cracow University of Technology, Warszawska 24, 31-155 Cracow, Poland; 2 Faculty of Civil Engineering, Cracow University of Technology, Warszawska 24, 31-155 Cracow, Poland; marcin.piechaczek1@doktorant.pk.edu.pl

**Keywords:** asphalt mix, polyethylene terephthalate, recycling, basalt filler, road pavements

## Abstract

Due to the growing need to recycle plastics, new possibilities for their reuse are intensively sought. In the Asian market, waste polymers are increasingly used to modify road bitumen. This solution is beneficial in many aspects, especially in economic and ecological terms. In this work, recycled poly(ethylene terephthalate) (RPET), obtained from storage points located in Lesser Poland, was subjected to material recycling, and its properties were examined using three analyses: differential scanning calorimetry (DSC), thermogravimetric analysis (TG), and Fourier transform infrared spectroscopy (FTIR). The most important point of this research was the selection of conditions for obtaining modified asphalt mixtures through the addition of RPET. Subsequently, the effect of the polymer on the properties of road bitumens was assessed on the basis of penetration tests, softening point, elastic recovery, and structure. In the last stage of our research work, asphalt mixtures with the addition of modified waste PET (PMA) containing mineral filler in the form of basalt dust were obtained. The properties of the obtained mineral–polymer–asphalt mixtures were compared in terms of frost resistance, structure, and abrasion resistance with the properties of mineral–asphalt mixtures that were taken from damaged road surfaces in four points in the city of Tarnów (Lesser Poland) in the winter of 2022. It has been shown that the modification of road bitumen with the use of recyclate and mineral filler has a significant impact on its performance properties.

## 1. Introduction

In the face of the impending climate crisis, it becomes necessary to reduce greenhouse gas emissions by utilizing renewable energy sources and limiting waste generation through the use of biodegradable materials and the improvement of waste processing methods. Therefore, important concepts in contemporary society in the early 21st century include carbon footprint reduction and the implementation of closed-loop principles for materials [1,2]. The awareness of this threat is confirmed by environmental protection guidelines introduced independently by the European Commission [3], the US government [4], India [5], and China [6,7]. One of the key pro-environmental demands mentioned in these guidelines is the reduction of plastic production and the development of effective recycling and processing methods for non-recyclable plastic waste. Currently, the most commonly recycled plastics due to their structure are PET (polyethylene terephthalate) and HDPE (high-density polyethylene) [8]. They can undergo mechanical or chemical recycling processes [9]. However, not all plastics are equally easy to process, and the recycling process is not always economically viable or widely available. Therefore, some plastics are less frequently recycled or not suitable for recycling.

**Figure 1:**
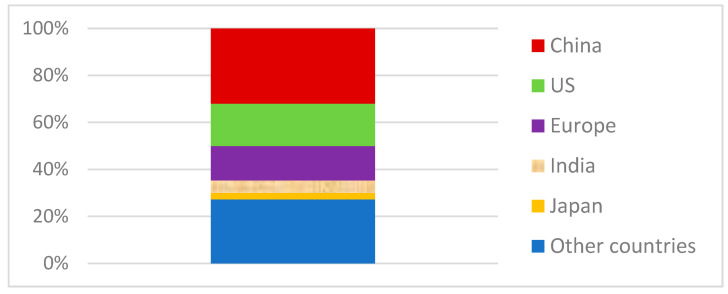
Share of countries in global plastic production in 2021 [11].

In 2021, global plastic production reached 390.7 million tons. China was the largest producer, accounting for 32% of this production, or approximately 125 million tons [10]. The United States ranked second, responsible for 18% of production, or about 70 million tons. Other leading producers include European countries, India, and Japan, with production amounts of approximately 57.2 million tons (15%), 21 million tons (5%), and 10.45 million tons (3%), respectively. The overall global plastic production overview is presented in Figure 1. According to forecasts, production by 2050 could exceed 600 million cubic meters [11].

Within the production of plastics, PET accounts for nearly 7% [12]. It is estimated that global PET production will reach 35.3 million tons in 2024, representing an increase of 4.8 million tons compared to 2019 [13]. According to [14,15], PET production in Japan in 2022 amounted to 358.97 thousand tons, which is an increase of 5.3 thousand tons compared to 2021. Referring to Figure 1, it can be observed that PET production worldwide is dominated by China, North and South America, and the rest of Asia [16].

Analyzing the area of plastic waste processing, it is estimated that in 2019, 353 million tons of waste were generated, of which 55 million tons were collected for recycling [17]. However, only 9%, i.e., 33 million tons, underwent recycling. This indicates deficiencies in the plastic waste-processing sector. In Europe, approximately 14% of plastics were recycled in 2019, while 37% were disposed of in landfills [18].

PET can undergo mechanical recycling, which involves washing, grinding, and melting it to produce new products [19,20]. However, the PET-recycling process leads to the breaking of polymer chains, resulting in a weakening of the polymer’s structure. Therefore, PET material can only be recycled 2–3 times before it becomes insufficiently durable for further use. In such cases, PET needs to be disposed of, meaning it is transformed into a different form than its original product. This process is called an open-loop system [21]. Waste PET plastic can be recycled within a closed-loop system in the textile industry [22], the packaging industry, as well as in construction. Ongoing efforts are being made to discover additional potential sources for PET recycling and utilization [23,24].

One solution within a closed-loop system appears to be the utilization of waste PET flakes for the preparation and production of asphalt mixtures. One potential application of waste PET flakes is their use in the preparation and production of asphalt mixtures within an open-loop system [22]. The use of additives in the production of modified asphalts is not a new concept, and the first attempts to modify bitumen date back to the early 1970s [25]. There are numerous possible modifiers used in asphalt mixtures, such as natural mineral fillers, which improve the flexibility of the mixtures and enhance the interaction between asphalt and aggregates. This is evidenced by studies described in [26,27].

The research conducted by Rieksts et al. in [28] presents an analysis of the rheological properties of sealing compounds with the addition of selected natural mineral fillers with various physical parameters. Physicochemical tests confirmed the effectiveness of using limestone fillers. In the work [26], Liu et al. presented the results of studies on modified asphalt mixtures using zeolite and limestone fillers. The conducted research indicated an improvement in the flexibility of the mixture by adding zeolite minerals while simultaneously reducing fatigue strength.

Guo and Chen, in [27], proposed the use of a limestone-based filler subjected to recycling. The results of their research indicate the potential for reusing the filler in asphalt mixture production. These studies aim to improve the performance and durability of asphalt mixtures while also promoting sustainable use of natural resources through material recycling.

Chemical compounds are also used to improve mechanical properties of asphalt. In [29], numerous examples of asphalt modification with chemical additives are presented. For instance, Siegmann’s study [30] is described, where asphalt was modified using chlorine. Chlorine modification reduced the penetration at 25 °C from 178 to 26 and increased the softening point from 39.5 °C to 82 °C. In another study, Edwards et al. [31] examined the influence of wax and polyphosphoric acid (PPA) modification on the rheological properties of asphalt binder and asphalt concrete mixtures. The study was conducted using a dynamic shear rheometer (DSR) and other conventional testing methods. The results indicate an increase in resistance to rutting for mixtures modified with commercial waxes or PPA. Yan et al. [32] analyzed the impact of PPA usage depending on the colloidal index of the asphalt. The results show an increase in the softening point and viscosity, especially for asphalts with a colloidal index greater than 2. Herrington et al. [33] demonstrated an increase in the softening point when maleic anhydride and dicarboxylic acids were used as modifiers for asphalt.

Rubber is a common additive used in asphalt production [34]. It improves resistance to rutting, aging, fatigue, and thermal cracking [35]. This is due to its structure, which consists of a liquid asphalt phase and swollen rubber particles [36]. Furthermore, the utilization of rubber from recycled used tires is also being considered [37,38]. Rubber can also be used to create bond coats. The formulation proposed by Zhang et al. [39] ensures integrity between the base and wearing-course layers. The application of the formulation resulted in a 30% increase in fatigue resistance.

Polymers are widely used as modifiers in asphalt production [40]. The most commonly used elastomers modified with copolymers are styrene-butadiene (SB), styrene-butadiene-styrene (SBS), and styrene-butadiene rubber (SBR). These polymers enhance asphalt’s resistance to extreme seasonal temperatures, improve adhesion between the binder and aggregates, and enhance resistance to rutting. These aspects can be attributed to a range of rheological effects. For example, Airey discussed the influence of SBS-modified asphalt on the rheology of mixtures in [41]. Rheological analysis was conducted using a dynamic shear rheometer. The results confirmed an increase in the mixture’s viscosity with higher polymer content. Numerous studies have demonstrated the effectiveness of SBS additives. These additives positively impact structural fatigue reduction and temperature reduction. The successful implementation of polymer-modified asphalts is evident through their commercialization in Poland [42] and other countries [43,44].

It is worth noting that despite modifications, binders and asphalt mixtures undergo aging processes. This process has been analyzed by Jacobs et al. in their work [45], where they described various protocols for the laboratory aging of binders and asphalt mixtures, using chemical–rheological properties.

There is also consideration for the reuse of modified polymer-modified bitumen (PMB) in road surface production. However, due to the aging processes that occur, research is being conducted on means to reverse the aging process of asphalt mixtures. Bajaj et al. [46] described the influence of rejuvenators on recycled asphalt mixtures. They emphasize that some compounds interact with the mixture solely at a physical level, while others affect physical parameters at a chemical level.

Margaritis et al. [47] conducted research on an asphalt mixture containing different percentages of recovered polymer-modified bitumen (RMPB) and a rejuvenating agent in the form of tall oil. Based on tests including a dynamic shear rheometer and infrared spectrometry, they found that the best results were achieved with a 20% addition of RMPB.

However, with the increasing awareness of recycling, the concept of using waste materials in asphalt production has emerged [48]. Waste materials such as steel slag, glass fibers [49], and polymer waste have been used for this purpose. The addition of waste aims to minimize the deterioration and deformation of road surfaces [50].

The concept of utilizing waste PET bottles aligns fully with current pro-environmental policies. Moreover, using polymer in asphalt production brings numerous benefits. Abuaddous et al. [51] prepared a series of samples based on asphalt with a penetration grade of 60/70, incorporating varying percentages of recycled polyethylene terephthalate (rPET). With an increase in the percentage of waste addition, the resistance to rutting improved. Simultaneously, there was an increase in high-temperature strength (from 64 to 70 for samples with 15% and 20% rPET, respectively). Fuentes-Audén et al. [52] described the influence of recycled polyethylene concentration on the rheological properties and microstructure of 150/200 penetration asphalt. As a result, incorporating 15% by weight of rPET as a modifier in the mixture improved the rheological properties. It is worth noting that the use of rPET led to a noticeable decrease in the glass transition temperature, resulting in better mechanical properties. Consequently, higher resistance to permanent deformation, reduced rutting, and increased resistance to thermal and fatigue cracking can be expected. Studies described in Polacco et al. [53] confirmed that polymer modification leads to the formation of a non-homogeneous structure that may undergo instability during mixture storage.

According to research conducted by Sharma and Sharma [54], modifying asphalt mixtures with plasticized asphalt additives (PCA) results in improved binding properties and reduced moisture. The modified samples exhibited a higher Marshall stability value within the range of 18–20 kN, representing a 100% increase compared to unmodified asphalt mixtures. The optimal percentage of plastic in the asphalt mixture, evaluated based on the Marshall stability test, was determined to be 10%. Similar results were obtained by Lugeiyamu et al. [55], who analyzed recycled PET. In accordance with the research conducted by Ma and Hesp [56], the influence of fibers made from processed PET as a modifier in asphalt mixtures was investigated to enhance the crack resistance. It was found that the use of longer and thicker PET fibers with a rough surface in asphalt mixtures at different temperatures can significantly improve their properties. Bekhedda and Merbouh [57] conducted research on asphalt mixtures modified with waste PET in two size variations and three mass contents (3%, 5%, 7%). The results showed that adding PET waste to the asphalt mixture can increase the resistance to permanent deformation and significantly improve the stiffness at various temperatures. The thermally more stable PET waste can also reduce the risk of degradation of bituminous mixtures. In studies conducted by Mashaan et al. [58], which utilized the Marshall stability test among others, the effect of adding waste PET and nanosilica to improve the resistance to rutting and fatigue of stone mastic asphalt (SMA) mixtures was examined. The results showed that adding 4–8% nanosilica in combination with 6% PET waste led to improvements in several key properties, such as resistance to rutting and reinforcement of the mixture’s stiffness modulus. The tensile strength and fatigue resistance were also improved, indicating greater durability of the material under dynamic stresses.

The work presented above shows the innovativeness of the ongoing research on the use of recycled PET and filler in the form of basalt meal to improve the performance of asphalt mixtures. This is influenced by the environmental aspect presented above.

Highly developed infrastructure is a basic feature of a highly developed city. Due to the need to carry out repairs to existing roads and plans to build new roads, this work attempts to develop a way to modify existing asphalt mixtures. For this purpose, waste PET was used, which came from waste collection sites in Tarnów. This is an excellent idea because it helps to solve not only the problem of waste management but is also an innovative way to reuse or extend the life of a product by changing its use. Modern cities may gain the title of “environmentally friendly cities” again because the addition of waste polyethylene terephthalate to asphalt surfaces will make them more environmentally friendly and their properties will be dramatically improved.

## 2. Research Concept and Analyzing Methods

The research work was divided into the following 4 stages:

Stage 1—Preparation of PET recyclates;

Stage 2—Characteristics of road bitumens;

Stage 3—Preparation of polymer-modified asphalt mixes (PMA);

Stage 4—Preparation of polymer-modified asphalt (PMA) mixes with the addition of mineral filler.

**Figure 2:**
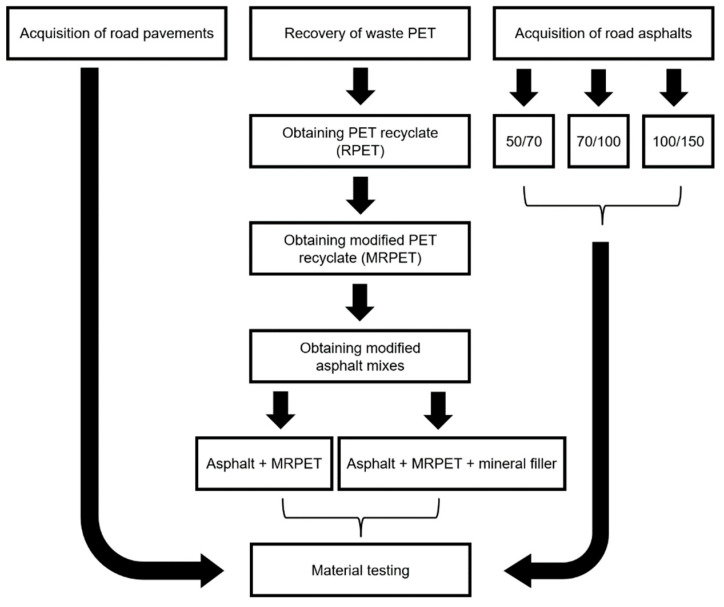
The schematic diagram of the research work.

Figure 2 presents a schematic diagram of the research work. 

### 2.1. Materials

Polyethylene terephthalate was obtained from bottles that were collected during selective waste collection in Lesser Poland (Poland). Waste PET was subjected to a grinding process using a Rapid-2b cutter designed for plastics, as a result of which recyclate (RPET) was obtained in the form of flakes.

The role of the RPET surface modifier was performed by a cationic emulsifier. Its manufacturer is ICSO Chemical Production Sp. z o.o. (Kędzierzyn-Koźle, Poland) and is marketed under the trade name Emulsamine^®^ L60.

Road bitumens of the following types were used: 50/70, 70/100, and 100/150, which were produced by ORLEN Asfalt Sp. z o. o. in Płock and Trzebinia (Poland) (Table 1).

Hydrochloric acid (HCl) was used in this work as a swelling component of modified asphalt mixtures. Hydrochloric acid (35–38% PURE), produced by POCH S. A. (Katowice, Poland), was used.

The role of the filler in the modified asphalt mixtures was played by basalt powder. The manufacturer of the filler used is NB Minerals (Tychy, Poland). Basalt fiber was also used as a filler in the modified asphalt mixtures. The manufacturer of the basalt fiber is Holtex S.A. (Lodz, Poland).

### 2.2. Methods

#### 2.2.1. Tests Performed on Polymers

DSC measurements were performed using a TOPEM^®^ DSC Mettler-Toledo 823e (METTLER TOLEDO, Warszawa, Poland) differential scanning calorimeter. The tests were carried out for the RPET and MRPET samples. The tests were performed according to the following temperature programs: first heating at 25–300 °C, first cooling at 25–300 °C, second heating at 25–300 °C and second cooling at 25–300 °C. The heating and cooling rate was 10 °C/min. 

TG measurements were performed using the NETZSCH TG 209F1 (Netzsch GmbH, Krakow, Poland) Libra apparatus. RPET and modified RPET (MRPET) samples were tested. The mass of RPET was 4.70 mg and that of modified RPET was 4.84 mg. The test was carried out in the temperature range of 30–65 °C, at a rate of 10 K/min, in an oxidizing atmosphere (15 cm^3^/min). 

In the FTIR study, the FTIR Spectrometer Nicolet iS5 (ThermoScientific, Waltham, MA, USA) was used. The spectrometer was equipped with an ATR iD7 (ThermoScientific, Waltham, MA, USA) diamond attachment. Measurements were made using the spectral range of 4000–400 cm^−1^ with 32 scans.

#### 2.2.2. Tests Carried Out for Road Bitumens, Polymer-Modified Asphalt Mixes, and Polymer-Modified Asphalt Mixes with the Addition of Filler

The penetration test provides information about the consistency of the bituminous material. The penetration test was carried out using an InfraTest penetrometer in accordance with the PN-EN 1426 standard [59]. The test was carried out for 5 s using a needle loaded with 100 g. The penetration vessel containing the bitumen sample was placed in a water bath at a temperature of 25 °C. Before the measurement, the sample was seasoned for 1.5 h in water at 25 °C.

The ring-and-ball softening point test allowed us to determine the temperature at which the bitumen began to liquefy. The automatic ring-and-ball apparatus type 20–2200 from InfraTest (infraTest Prüftechnik GmbH, Brackenheim, Germany) was used to measure the softening point. The measurement was carried out in accordance with the PN-EN 1427 standard [60]. Distilled water was used as the heating medium. Before measurement, bitumen was poured into two brass rings. Then, the sample was seasoned until the temperature reached 5 °C. The rings were placed sequentially with asphalt and guides with balls in the apparatus stand and then in the beaker with the heating medium.

The elastic recovery test was carried out using a digital ductometer type 20-2346 by InfraTest, in accordance with the PN-EN 13398 standard [61]. The measurement was carried out in a tub of the ductometer filled with water at a temperature of 25 °C. Before proceeding with the measurement, samples of liquid asphalt were poured into special molds. Subsequently, the samples were seasoned for 60 min in the same conditions in which the measurement was performed.

A frost resistance test was carried out at −10 °C for 48 h. Before starting the test, samples of asphalt mixes collected in Tarnów and samples of modified asphalt mixes were soaked in a bath of NaCl salt at a concentration of 40 wt% for 24 h.

The determination of abrasion was carried out using KL 381 J 60 sandpaper from Klingspor (Klingspor, Bielsko-Biala, Poland), which was attached to a metal drum in accordance with the PN-69/C-89081 standard [62]. The force of pressing the sample to the paper ranged from 10 to 30 N. Prior to testing, samples of asphalt mixes collected in Tarnów and samples of modified asphalt mixes were attached to cork holders and weighed on an analytical balance. In order to determine the abrasion value, the density of the tested samples was determined using the pycnometric method.

The study of the structure of road bitumens, polymer-modified asphalt mixtures, and polymer-modified asphalt mixtures with the addition of filler was carried out using a metallographic microscope with a Delta Optical MET-200 camera (Delta Optical, Gdansk, Poland). The analysis of the structure of the tested material was carried out with image magnifications of 4×, 10×, and 40×.

## 3. Preparation of PET Recyclates

Polyethylene terephthalate was used as an essential component of polymer-modified asphalt mixtures. The collected waste showed various properties, e.g., color and stiffness. Prior to proceeding to the key phase of work, the collected polyester was cleaned and modified by means of material recycling.

The next step consisted of removing caps, most often made of polypropylene (PP) and polyethylene (PE), and polyvinyl chloride (PVC) labels. This was necessary since their presence would prevent the fragmentation of bottles into smaller pieces. Furthermore, the plastics from which they were made could have a substantial impact on the physicochemical and mechanical properties of waste polyethylene terephthalate, e.g., a reduction in molecular weight owing to degradation. 

The next step of material recycling involved the use of a Rapid-2b slitter, which made it possible to crush PET bottles into small flakes with dimensions from 3 to 5 mm. For cleaning purposes, the PET flakes were soaked in a 3 wt% aqueous sodium hydroxide (NaOH) solution. Furthermore, the flakes were treated with three rinses in hot water at 90 °C.

The last step in the preparation of waste polyethylene terephthalate was its surface modification with the cationic emulsifier Emulsamine^®^ L60. The excess emulsifier was removed by drying at room temperature for about a month. The above-mentioned steps resulted in obtaining a modified recyclate (RPET), which was subjected to injection-molding and extrusion processes and used to modify asphalt mixtures.

Samples were created from recycled PET (RPET), mechanical recycled PET (MRPET), and PET granulate in the form of a processed material, both by means of extrusion and injection molding.

### 3.1. Characteristics of PET and PET Recyclates

In order to verify the physicochemical and thermal properties of the obtained PET varieties, the following studies were carried out: differential scanning calorimetry (DSC), thermogravimetric analysis (TG), and Fourier transform infrared spectroscopy (FTIR).

#### 3.1.1. Differential Scanning Calorimetry (DSC)

**Figure 3:**
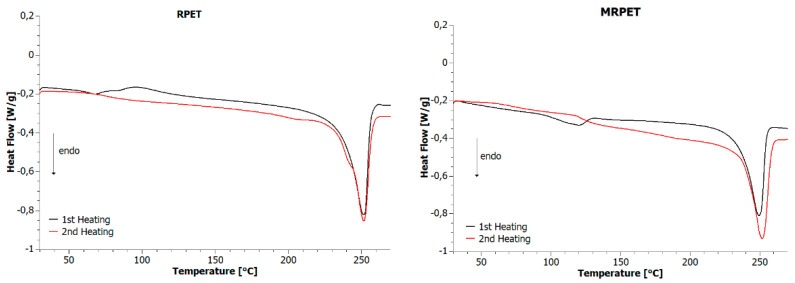
DSC curves obtained during the first and second heating programs for RPET and MRPET.

Differential scanning calorimetry tests were carried out for RPET and MRPET samples using the same temperature programs. The analysis of DSC curves (Figure 3) for the first and second heating programs provided the following information: during the first heating program, in the case of the MRPET sample, there was a glass transition in the temperature range of 100–150 °C, while the RPET sample was subjected to this transformation in the temperature range of 50–100 °C. When considering the effects achieved by analysis of the results for the second heating program, the transition was gentler in the same temperature ranges for both materials. For the first and second heating curves, a clear endothermic peak, which is characteristic of the melting process, can be noted for both samples. It is pertinent to note that the apex of the endothermic peak (during the second heating program) for both the RPET and MRPET samples was present at the same temperature. The peak intensities varied by approximately 0.1 W/g. For RPET, it was −0.85 W/g while, for MRPET, it was −0.95 W/g. The shape of DSC curves obtained during the second heating program for the RPET and MRPET samples varied. 

**Figure 4:**
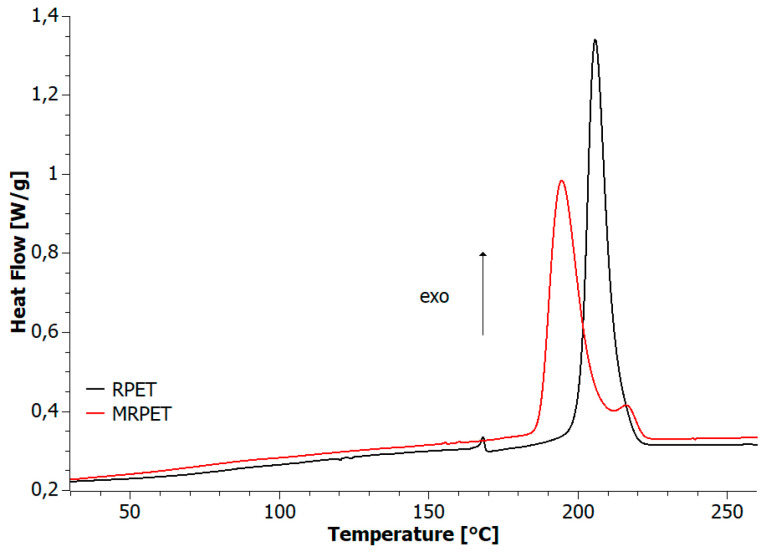
DSC curves for RPET and MRPE samples’ cooling programs.

In the case of the DSC cooling curves for the RPET and MRPET samples, clear, sharp peaks characteristic of a fast exothermic process such as crystallization were observed (Figure 4). The peak intensity of the crystallization process was 1.35 W/g for RPET and 0.98 W/g for MRPET.

#### 3.1.2. Thermogravimetric Analysis (TG)

RPET and MRPET samples were subjected to thermogravimetric analysis (TG) in order to examine the influence of recyclate modification on the degradation process.

**Figure 5:**
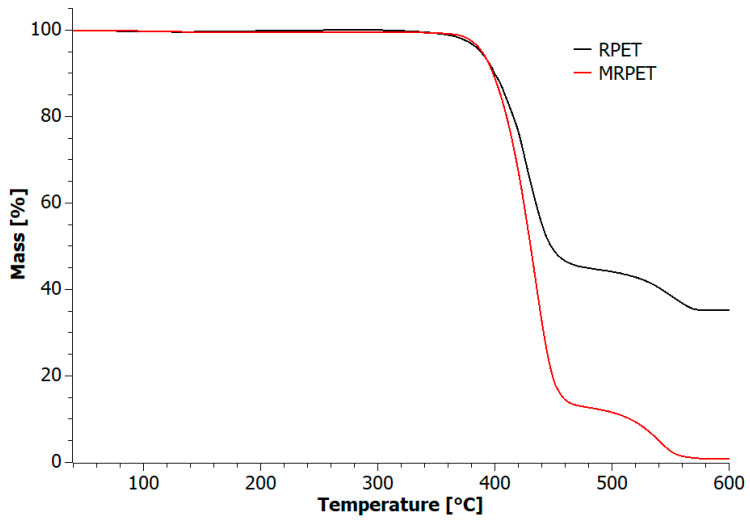
TG curves of the investigated materials during thermooxidative degradation.

TG analysis (Figure 5) demonstrated that the thermal decomposition process involved two steps for both samples. The MRPET sample had a significantly greater weight loss than the RPET sample. The modified recyclate underwent 85% decomposition at 480 °C, while the RPET sample lost only 45% of its original weight under the same conditions. On the basis of the obtained DTG curves, the temperature of the maximum weight loss was determined, i.e., 424 °C for RPET and 434 °C for MRPET.

#### 3.1.3. Fourier Transform Infrared (FTIR) Spectroscopy

The Fourier transform infrared (FTIR) spectroscopy study’s aim pertained to the examination of the influence of processing methods, namely injection molding and extrusion, on the properties of RPET and MRPET samples. For the purposes of comparison, pure PET granules were also tested. 

**Figure 6:**
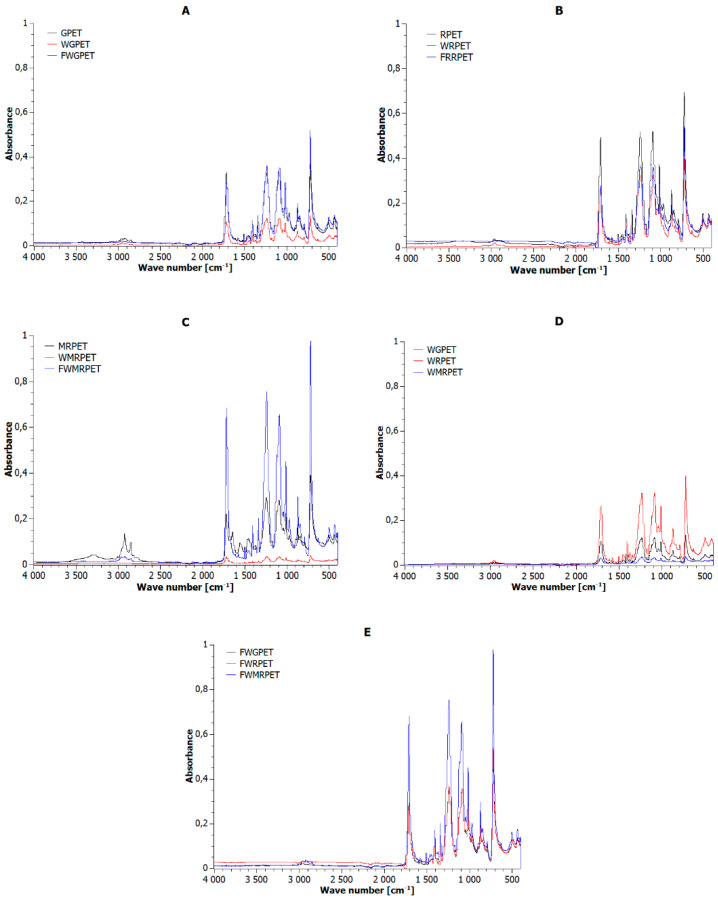
FTIR spectra for: (**A**) PET granulate, (**B**) PET recyclate, (**C**) modified PET recycalate, (**D**) granulate, recyclate, and modified PET recyclate subjected to the extrusion process, (**E**) granulate, recyclate, and modified PET recyclate subjected to the injection-molding process.

Based on the analysis of the spectra (Figure 6), it was found that the individual samples revealed the same absorption bands but of differing intensity. The changes occurred for the MRPET sample, for which the spectrum showed an absorption band at the wavenumber value of 3300 cm^−1^ and a much greater intensity of the absorption band at wavelengths of 2800 and 2900 cm^−1^.

The IR spectrum of PET granulate had low-intensity bands, corresponding to vibrations stretching the O–H bond in the carboxyl group, located at the wavenumber value of approx. 2900 cm^−1^. Another band, with an intensity of approx. 0.4, occurring for the wavenumber 1710 cm^−1^, was assigned to the stretching vibration in the C=O bond of the carbonyl group which is present in the ester bond and carboxyl end groups. The presence of C=C stretching frequencies in the aromatic ring was associated with the appearance of the absorption band at 1414 cm^−1^. There were also absorption bands with this intensity at wavenumber values of 1010, 1100, 1241 cm^−1^ in the spectrum, which corresponded to the stretching vibrations in the C–O functional group present in the esters. The spectrum also showed an absorption band of medium intensity, approx. 0.2, which appeared at a wavelength of 800 cm^−1^. They were assigned to the stretching vibrations in the =C–H group present in the aromatic ring from polyethylene terephthalate. The spectrum also showed stretching vibrations for the C–H bond, present in the aromatic PET ring, which corresponded to the absorption band at the wavenumber values of 710 and 1350 cm^−1^. 

The intensity of absorption bands in the IR spectrum of the modified RPET recyclate was most diversified. The absorption bands for modified RPET subjected to the injection-molding process were characterized by the highest intensity. 

In turn, the extruded MRPET sample exhibited the lowest intensity of absorption bands. Moreover, the presence of an absorption band found only in the spectrum of the MRPET sample was observed at a wavenumber value of 3300 cm^−1^. This band corresponded to the vibration stretching the N–H bond present in the ammonium ion of the cationic emulsifier used to modify the RPET granulate. The spectrum also showed high-intensity absorption bands at wavelengths of 2900 cm^−1^ and 2800 cm^−1^, which were attributed to symmetrical and asymmetric stretching vibrations in the NH^3+^ group, which is likely to exist in the RPET modifier. 

The intensity of the absorption bands of the extruded samples was characterized by an enormous disparity. The WRPET sample showed the highest absorption intensity of infrared radiation, while the WMRPET sample showed the lowest. 

The samples obtained by injection molding FWGPET and FWRPET had the same intensity of absorption bands across the entire spectral range, while in the case of FWMRPET, the intensity was twofold higher.

## 4. Properties of Road Construction Bitumen

### 4.1. The Influence of Temperature on the Penetration Value of Road Construction Bitumen

**Figure 7:**
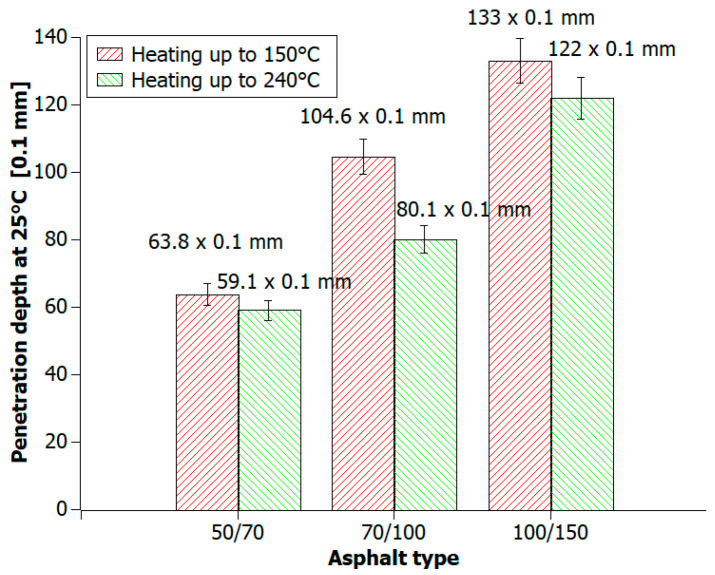
Road bitumen (50/70, 70/100, and 100/150) penetration values heated at temperatures of 150 °C and 240 °C.

The objective of this study was to verify how heating bitumen at 150 °C and 240 °C affects its penetration value (Figure 7). The study covered three types of road construction bitumen: 50/70, 70/100 and 100/150. Penetration for each type of bitumen decreased slightly. The greatest decline was related to 70/100 bitumen and it was approx. 25 × 0.1 mm. In the case of 50/70 and 100/150 bitumen, the decrease in the penetration value was 4 × 0.1 mm and 10 × 0.1 mm, respectively.

### 4.2. The Impact of Heating Road Construction Bitumen on the Softening Point Value Measured by the Ring-and-Ball Method

The effect of high temperature on bituminous materials causes changes in their consistency: the bitumen begins to liquefy, which can damage the asphalt pavement. Determination of the softening point allows one to establish the places in which a given type of bitumen can be used, e.g., car parks or road asphalt surfaces. During our research work, 50/70, 70/100, and 100/150 road construction bitumen had to be heated at 240 °C in order to obtain polymer-modified bitumen mixtures. Therefore, the softening point was measured by the ring-and-ball method for the three aforementioned types of bitumen in order to investigate the effect of heating bituminous materials at 150 °C and 240 °C. 

**Figure 8:**
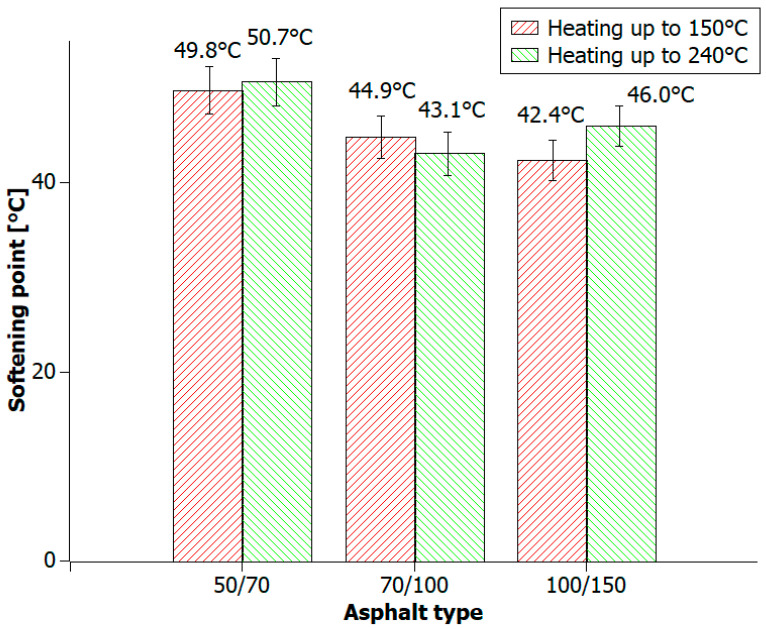
The softening point values of road bitumens (50/70, 70/100, and 100/150) heated at temperatures of 150 °C and 240 °C.

The analysis of measurement results (Figure 8) of the softening point showed (as in the previously discussed measurement results) that heating bitumen has merely a small impact on the tested value. The 50/70 bitumen, heated at 240 °C, had a higher softening point value by about 1 °C compared to the same bitumen heated at 150 °C. The softening point difference for the 70/100 bitumen was also 1 °C. At the temperature of 240 °C, 100/150 bitumen showed an approx. 4 °C higher softening point than at 150 °C.

### 4.3. The Value of Elastic Recovery of Road Construction Bitumen Subjected to High Temperature

If a rut is formed in an asphalt road surface, then using road construction bitumen with a high recovery value allows one to return the bituminous material to its original form. During the study, elastic recovery tests were carried out for 50/70, 70/100, and 100/150 bitumen heated at 150 °C and 240 °C in order to verify the presence of elastic properties and the influence of temperature on elastic properties.

The results confirmed that road construction bitumen did not exhibit elastic properties (Table 2). Moreover, none of the bitumen grades showed elastic recovery above 18%. It is noteworthy that the manufacturer of the bitumen used did not present the value of elastic recovery for road construction bitumen in any sources, because a high value of elastic recovery is characteristic solely for polymer-modified products and is at a level of over 50%.

## 5. Preparation of Polymer-Modified Asphalt (PMA) Mixtures

The process of preparation of polymer-modified asphalt (PMA) was applied to three types of road construction bitumen: 50/70, 70/100, and 100/150. 

In step one, each type of bitumen was heated in a HORYZONT Spt 200 dryer (Zeamil Horyzont, Krakow, Poland) to a temperature of about 150 °C. As soon as a liquid consistency was obtained, bitumen was divided into several smaller portions weighing about 300 g. For this purpose, metal cans with resistance to very high temperatures were used. The modification process was started by heating the bitumen to a temperature of 240 °C for about 60 min. 

After the bitumen had reached the set temperature, the emulsifier-modified MRPET was added in three different amounts: 2, 5, and 10 wt%. After the addition of polyester, the bitumen was heated at 240 °C for about 15 min. In the next step, the prepared polymer-modified asphalt composition was mixed with an R50D CAT stirrer (Caterpillar Inc, Irving, TX, USA) at a speed of 800 rpm for about 7 min. When the mixture obtained a homogeneous consistency, a solution of hydrochloric acid with a concentration of 10 mol/dm^3^ was added. The solution was prepared in advance by appropriate dilution of hydrochloric acid with 35–38% CZDA. During the addition of the acid, foaming as well as swelling of the bitumen were observed. Next, the finished polymer-modified asphalt mixture was cooled at room temperature until the sample solidified within the entire volume of the vessel. The PMA preparation process outlined above obtained nine samples (Table 3).

### 5.1. Penetration Depth of Polymer-Modified Asphalt (PMA)

In order to verify the effect of the addition of MRPET to asphalt on the penetration depth of road construction bitumen heated to 240 °C, the obtained polymer-modified asphalt mixture was subjected to penetration tests. 

**Figure 9:**
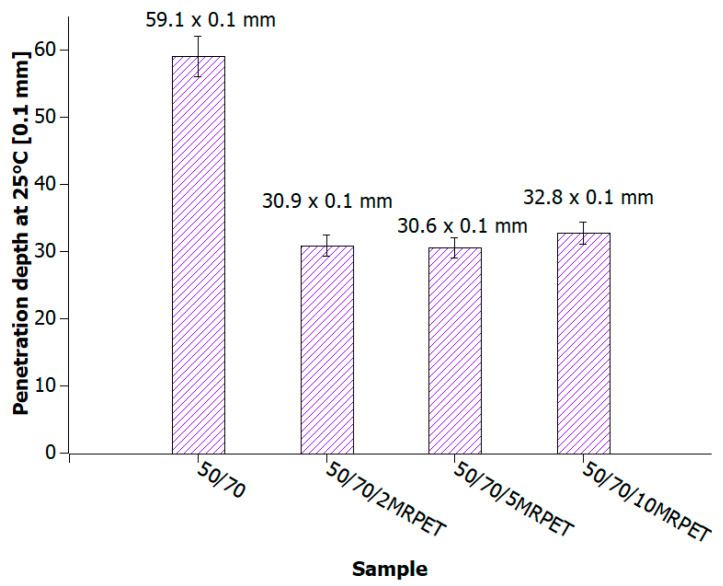
Penetration values of 50/70 bitumen heated to 240 °C and successively with 2, 5, and 10 wt% MRPET.

Penetration of 50/70 bitumen after adding MRPET decreased by about 30 × 0.1 mm for each of the amounts of modified recyclate used. The penetration value increased slightly with increasing MRPET content (Figure 9). 

**Figure 10:**
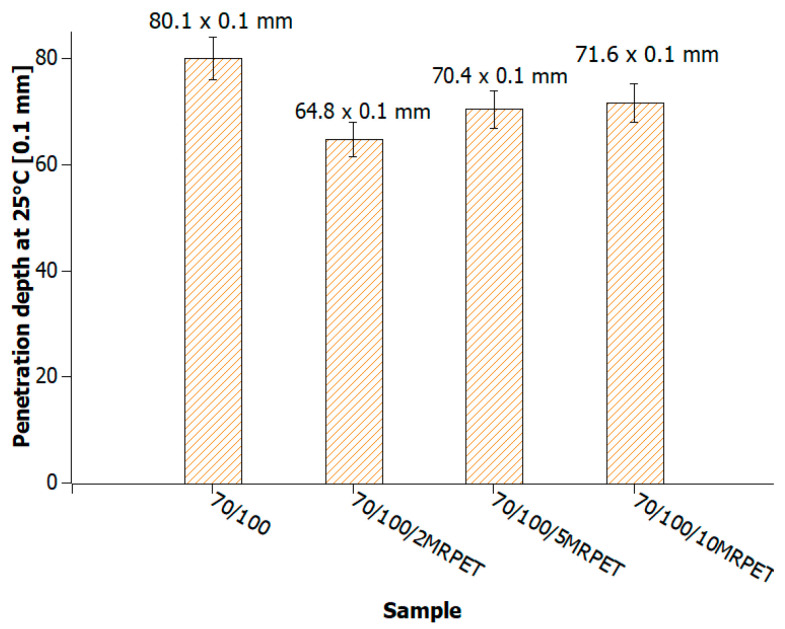
Penetration values of 70/100 bitumen heated to 240 °C and successively with 2, 5, and 10 wt% MRPET.

The penetration of 70/100 bitumen was slightly lower than that of the other bitumen grades due to the apparent instability of the samples. The penetration of 70/100 bitumen in the case of the addition of 2 wt% MRPET decreased by about 15 × 0.1 mm and, for 5 wt% and 10 wt%, by about 10 × 0.1 mm (Figure 10). 

**Figure 11:**
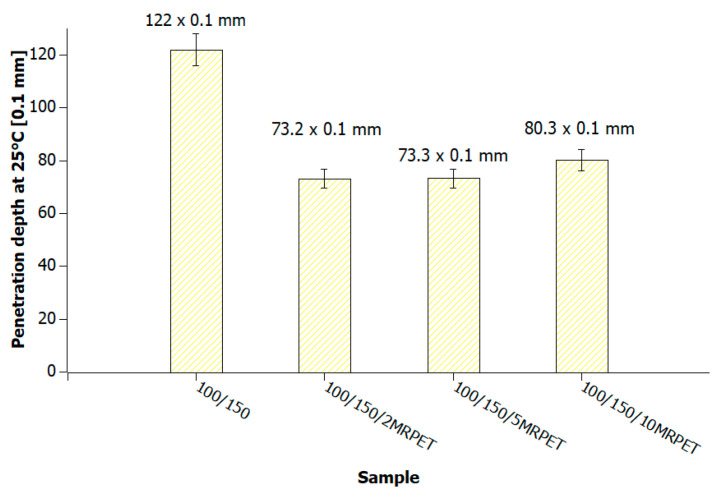
Penetration values of 100/150 bitumen heated to 240 °C and successively with 2, 5, and 10 wt% MRPET.

The reference penetration of 100/150 bituminous material with a value of 122 × 0.1 mm decreased by about 50 × 0.1 mm due to the addition of 2 and 5 wt% MRPET. The addition of 10 wt% recyclate caused a decrease in the tested value by about 40 × 0.1 mm compared to the reference sample. Due to the addition of MRPET to 100/150 bitumen, its penetration was reduced to about 65 × 0.1 mm (Figure 11).

The penetration of 70/100 and 100/150 bitumen decreased slightly with the high addition of polyester, reaching 10 wt%.

### 5.2. The Softening Point of Polymer-Modified Asphalt (PMA) by the Ring-and-Ball Method

**Figure 12:**
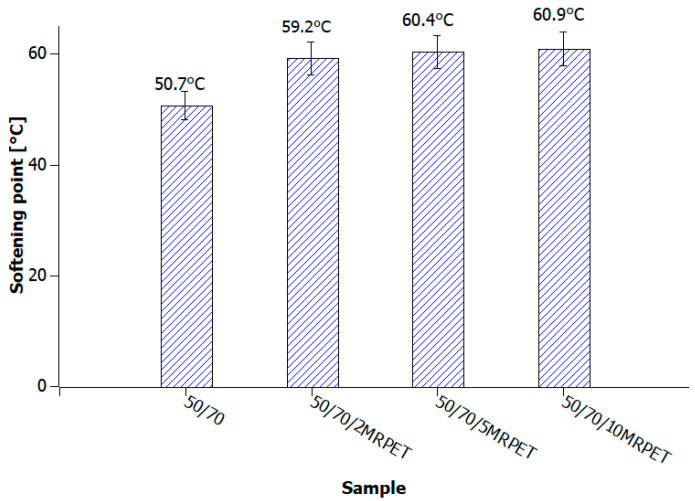
The values of the softening point of 50/70 bitumen heated to 240 °C and successively with the addition of 2, 5, and 10 wt% MRPET.

**Figure 13:**
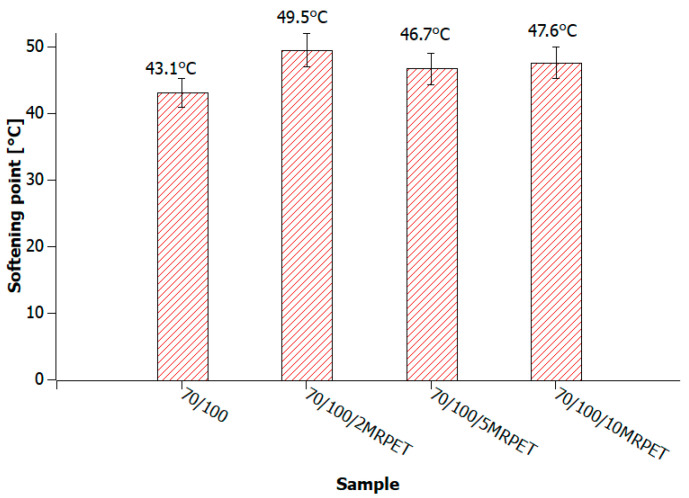
The values of the softening point of 70/100 bitumen heated to 240 °C and successively with the addition of 2, 5, and 10 wt% MRPET.

**Figure 14:**
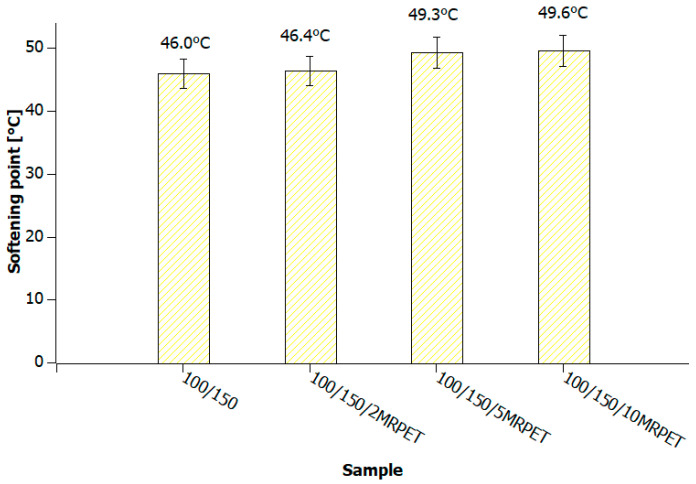
The values of the softening point of 100/150 bitumen heated to 240 °C and successively with the addition of 2, 5, and 10 wt% MRPET.

The analysis of the results demonstrates that in the case of polymer-modified asphalt, in which the base bitumens were grade 50/70 and 100/150, the tested value increased slightly with the addition of MRPET (Figure 12, Figure 13 and Figure 14). 

In the case of the 70/100 bitumen with the addition of RPET, there was no orderly upward trend observed due to the instability of the sample.

### 5.3. The Elastic Recovery of Polymer-Modified Asphalt (PMA)

It was expected that the obtained polymer-modified asphalt (PMA) would obtain elastic properties, which are valuable in road construction, as a result of the addition of MRPET. For this purpose, they were subjected to studies of elastic recovery (Table 4).

In the case of 50/70 bitumen, the addition of 2, 5, and 10 wt% MRPET increased the value of elastic recovery by 6, 10 and 20%, respectively. The addition of modified recyclate in amounts of 2 and 5 wt% for 70/100 and 100/150 bitumen did not have an appreciable effect on the value of elastic recovery. Moreover, the addition of 2 wt% MRPET reduced the value of elastic recovery for both types of bitumen.

### 5.4. Study of the Structure of Polymer-Modified Asphalt (PMA)

Polymer-modified asphalts can form three different types of dispersion. It was expected that the obtained polymer-modified asphalt (PMA) would have a similar structure. In order to verify the influence of MRPET additive on the structure of road construction bitumen, 50/70, 70/100, and 100/150 bitumens were analyzed microscopically. At a later stage, they constituted the reference samples and polymer-modified asphalt (PMA). The study was expected to provide detailed information on polymer-modified mineral mixtures. 

The 50/70 bitumen reference sample had numerous losses in its structure (Table 5). The addition of MRPET caused a shredded surface of the bituminous material sample. This significantly disrupted the microscopic observation of the sample in reflected light. The addition of 2 wt% recyclate contributed to a significant deformation of the bitumen. The microscopic image shows only black areas with glowing peaks. By adding 5 wt% MRPET, the structure had a much greater number of cavities compared to the reference sample. Samples with the addition of modified recyclate in an amount of 10 wt% had numerous cracks in the structure.

The microscopic photo of the 70/100/2RPET sample shows a misfolded bitumen surface with a few areas of partial weight loss (Table 6). Modification of 70/100 bitumen with 5 and 10 wt% RPET contributed to the creation of numerous holes in bituminous material. In addition, sample 70/100/10RPET was characterized by an undulating structure. 

The addition of 2 wt% RPET to 100/150 bitumen caused a loss of uniform surface due to the formation of micropores. Sample 100/150/5RPET had a porous structure.

Clusters of recyclate could be observed in the microscopic image of the 100/150/10RPET sample (Table 7). The mode of operation of RPET resulted in the destruction of the bitumen mass in certain places. The structure was heavily perforated in some areas, cracked as a result of the recyclate, and in some places, there were clusters of increased amounts of polymer phase. In the remaining areas, the surface of 100/150 bitumen remained smooth.

## 6. Preparation of Polymer-Modified Asphalt (PMA) with the Addition of Mineral Filler

The results of the cumulative measurements carried out for polymer-modified asphalt mixtures (PMA) showed that the addition of 10 wt% MRPET allowed us to obtain stable penetration results and the highest values of the softening point as well as to increase the elastic recovery value by nearly 20%.

Based on the analysis of the results of the above measurements, it was decided that the addition of modified RPET in polymer-modified asphalt with filler would be 10 wt%. This choice was made in view of the fact that the content of MRPET did not have any significant bearing on the value of the parameters tested; therefore, for economic and ecological reasons, polymer-modified asphalt mixtures with mineral filler with a content of 10 wt% MRPET were obtained.

Polymer-modified asphalt mixtures were obtained with the use of 10 wt% MRPET and 50 wt% mineral filler. The choice of the amount of mineral filler in the polymer-modified asphalt composition was dictated by the fact that due to the addition of MRPET, the polymer-modified asphalt (PMA) mixtures achieved penetration values characteristic of road construction bitumen used as components of SMA mixes, in which the content of mineral aggregate is 60–80%. In the obtained polymer-modified asphalt mixtures, the value of this parameter was reduced by 10% through the addition of MRPET, thanks to which they achieved a high degree of homogenization with the mineral component distributed in them.

The function of mineral fillers in polymer-modified asphalt mixtures with mineral filler was played by basalt flour (BFL) and basalt fiber (BFB).

In view of the fact that the paddle stirrer could not reach a speed of 800 rpm (its rotation was inhibited by the basalt fibers and the consistency of the bituminous material was too viscous: the stirring process was not sufficient to achieve a homogeneous consistency of the material), the samples with basalt fiber were not subjected to further testing.

As a result of the above-described process for the preparation of polymer-modified asphalt mixtures with mineral filler, six samples were obtained (Table 8).

### 6.1. Characteristics of Polymer-Modified Asphalt Mixtures (PMA) with the Addition of Mineral Filler

The properties of the polymer-modified asphalt mixtures with mineral filler were compared with the properties of samples of mineral-modified asphalt mixtures, which were collected from four sensitive areas located in the city of Tarnów, Lesser Poland (50°00′49″ N, 20°59′13″ E). The above-mentioned samples were subjected to tests determining resistance to negative temperatures, microscopic analysis of the structure, and abrasion tests. This research aimed to explore whether the properties of the obtained polymer-modified asphalt mixtures with mineral filler were so favorable that they could replace the mineral-modified asphalt mixtures used so far for the construction of asphalt road surfaces in the city of Tarnów (Table 9).

#### 6.1.1. Testing Resistance to the Effect of Negative Temperature—Frost Resistance of PMA Mixtures with Mineral Filler and Mineral-Modified Asphalt Mixtures from Tarnów

Asphalt pavements in Poland are subject to negative temperatures. As a result of the water freezing inside the asphalt layer, they burst. Polymer-modified asphalts show high resistance to cracking due to the elastic properties provided by the addition of a polymer. In order to verify the resistance of the obtained polymer-modified asphalt mixtures with mineral filler, they were subjected to a frost resistance test. Furthermore, the test was also carried out for samples of polymer-modified asphalt mixtures from Tarnów in order to compare their frost resistance properties with those of PMA. 

The analysis of photographs (Table 10) provided more information about the resistance of the tested samples to negative temperature. The frost resistance test demonstrated that the obtained polymer-modified asphalt mixtures with mineral filler have a similar resistance to the effect of negative temperature in relation to the mineral-modified asphalt mixtures collected from the streets of Tarnów. Samples with 10 wt% MRPET had no cracks. Crystallized salt was observed on samples from the city of Tarnów.

#### 6.1.2. Examination of the Structure of PMA Mixtures with Mineral Filler and Mineral-Modified Asphalt Mixtures from Tarnów

In order to investigate the effect of the addition of mineral filler to the polymer-modified asphalt mixtures and to compare the structure of the obtained PMA mixtures with the addition of basalt dust with that of mineral-modified asphalt mixtures (MMA) from Tarnów, a further microscopic analysis was carried out. It was expected that as a result of the addition of mineral filler to PMA mixtures, they would obtain a similar structure to that of the MMA samples from Tarnów. 

The microscopic analysis (Table 11) of the samples was particularly difficult. The reason was the uneven surface of the research material and the presence of aggregate remnants. The structure of the polymer-modified asphalt mixtures with mineral filler was similar to that of the mineral-modified asphalt mixtures from Tarnów. The most notable changes could be seen between the 50/70/10MRPET/PB sample and the other samples from the Tarnów area. The mineral filler was highly dispersed inside the asphalt layer. Dispersions of two materials were obtained. There was one dominant phase therein, but there were two interpenetrating phases—the asphalt phase and the mineral phase. The microscopic image of the 100/150/10MRPET/PB sample had elements of a very regular shape which could suggest that they were salt crystals.

#### 6.1.3. Test of the Abrasion of Polymer-Modified Asphalt Mixtures with Mineral Filler and Mineral-Modified Asphalt Mixtures from Tarnów

Asphalt pavements are often damaged due to insufficient abrasion resistance. It was expected that adding MRPET to road construction bitumen would improve this parameter. For this purpose, the polymer-modified asphalt mixtures with mineral filler were subjected to an abrasion test. The test was also performed for mineral-modified asphalt mixtures from Tarnów. This enabled the comparison of their abrasion resistance.

70/100/10MRPET/PB and 100/150/10MRPET/PB had greater abrasion resistance than the bituminous mixtures collected in the city of Tarnów (Table 12). The abrasion of 50/70 bitumen with the addition of 10 wt% MRPET and mineral filler, the value of which was 6.56 mm^3^/m, was similar to the abrasion value for MMAM, MMAK, and MMAMR samples, which ranged between 6 and 9 mm^3^/m. Due to the numerous damages to sections of these roads, it could be assumed that they did not exhibit high abrasion resistance. The use of 70/100 and 100/150 bitumens as base bitumen and MRPET as a polymer additive made it possible to obtain an asphalt mix with high abrasion resistance.

## 7. Discussion

The first stage of this research comprised obtaining samples of mineral–asphalt mixtures from the surface of damaged roads located in Tarnów. In the course of experimental research, appropriate conditions for modifying road bitumens were selected. Asphalt mixtures with the addition of MRPET and, in a further step, mineral filler were obtained. The conducted research showed that the performance and strength properties of the obtained mixes depended primarily on the type of base bitumen used.

The properties of the modified RPET were investigated using three analyses: differential scanning calorimetry (DSC), thermogravimetric analysis (TG) and Fourier transform infrared spectroscopy (FTIR). Thermogravimetric analysis (TG) showed that the modified RPET was degraded at a temperature of about 400 °C. During the preparation of polymer-modified asphalt mixtures (PMA), MRPET was subjected to 240 °C, so the developed conditions of the PMA preparation method did not cause thermal decomposition of the modified recyclate. On the DSC curve for the MRPET sample, the melting process started at 235°, so due to the conditions used in the developed method of obtaining PMA, the MRPET was completely melted. Fourier transform infrared spectroscopy (FTIR) showed that the MRPET sample had a higher intensity of infrared absorption for individual functional groups in relation to the RPET and PET samples, and absorption bands were observed in its IR spectrum, which corresponded to bond vibrations occurring in the cationic emulsifier.

Polymer-modified asphalt (PMA) mixtures were tested for penetration, softening point, elastic recovery, and structure. The addition of modified PET recyclate had the greatest impact on the penetration values of the base bitumens. As a result of the addition of MRPET to the 50/70 base bitumen, the penetration decreased to 30 × 0.1 mm. The addition of MRPET to the 50/70 bitumen made it reach the degree of penetration, which is shown by polymer-modified bitumens produced by Orlen Asfalt Sp. z o. o. (Orlen S.A., Chorzów, Poland) (the main producer in Poland). By adding RPET to the 100/150 asphalt, a penetration rate of 70 × 0.1 mm was achieved, thanks to which the obtained polymer-modified asphalt mixture could act as a component of SMA mixtures. The 70/100 asphalt used as a base showed high instability; therefore, the addition of RPET did not significantly affect its penetration. The softening point values of all polymer-modified asphalt mixtures slightly increased, thanks to which the PMAs gained greater rutting resistance under the influence of high-temperature loads on the surface of asphalt pavements. The elastic recovery test showed that the MRPET addition did not impart elastic properties to the bitumen. Based on the study of the PMA structure, information was obtained on how MRPET affects the structure of the asphalt mass. Numerous holes could be seen in the microscopic photographs of the base bitumens. The addition of MRPET resulted in significantly more cavities in the bitumen. The material was classified as a polymer–asphalt dispersion, in which there was a continuous polymer phase with a dispersion of asphalt particles. As a result of the modification, the asphalt mass became porous and corrugated. The porosity was increased by the addition of 2, 5, and 10 wt. polyester. MRPET also showed a punctual effect. In some parts of the bituminous material, clusters of modified recyclate could be seen.

The polymer-modified asphalt mixtures with 10% MRPET, selected for the best properties, were filled with basalt dust amounting to 50% by weight. Obtained mineral–polymer–asphalt mixtures together with samples of mineral–asphalt mixtures taken from asphalt pavements in the city of Tarnów (Lesser Poland) were subjected to several utility tests, i.e., tests of resistance to low temperature—frost resistance, structure tests, and abrasion tests. The frost resistance test showed that the modified mineral–polymer–asphalt mixtures were characterized by the same resistance to negative temperatures as the samples collected in the city of Tarnów. The study of the structure showed that the structure of all obtained samples of mineral–polymer–asphalt mixtures and mineral–asphalt mixtures collected in Tarnów was similar. The photos obtained as a result of the microscopic analysis showed the dispersion of two phases—asphalt and mineral. Based on the analysis of the abrasion test, it was found that the polymer-modified asphalt mixes obtained on the basis of 70/100 bitumen showed the highest abrasion resistance. Thanks to this, they could be used as components of the wearing course of asphalt pavements.

## 8. Conclusions

Based on the obtained results, it can be concluded that the modification of road bitumens with the use of recyclate and mineral filler has a large impact on their performance properties. The resulting polymer-modified asphalt (PMA) mixtures can be used in wearing courses due to their high abrasion resistance and for the construction of intersections and parking areas due to the reduced penetration value. The modification of road asphalts with the use of waste PET is an excellent solution economically and ecologically. Due to the increasing production of polyethylene terephthalate, the search for new solutions for its reuse is the key to the development and implementation of material recycling in line with sustainable development.

The properties of road bitumens of grades 50/70, 70/100, and 100/150 can be significantly changed and improved by modification. In the obtained polymer-modified asphalt mixes, they acted as base bitumens. They contained RPET modified with a cationic emulsifier, which was used in various amounts of 2, 5, and 10% by weight, as well as 10 M hydrochloric acid in an amount of 2.5% by weight.

Looking at the conclusions of this experimental work, it can be expected that the production of polymer-modified asphalt mixtures in the future will be beneficial both in economic and ecological terms.

At the moment, polymer-modified asphalts are of great interest due to their unique properties. Although mixtures containing copolymer components (even recycled ones) prevail on the market, currently, their biggest disadvantage is the high production price. The results described in this paper open new avenues through the use of waste PET, which until now seemed practically impossible. It is worth noting that the advantage of this solution is the reduction in financial outlays needed to purchase the polymer. Cities, by using an asphalt mixture containing MRPET in street fragments, driveways, parking lots, and even sports fields, would gain asphalt surfaces of excellent quality and would save a significant part of their budget by reducing the costs associated with road repair.

## 9. Future Perspectives

Looking at the conclusions resulting from this experimental work and the literature review, it can be expected that the production of polymer-modified asphalt mixtures will be beneficial both economically and ecologically for the city of Tarnów in the future. Polymer-modified asphalts are currently receiving great interest due to their unique properties. Although the market is dominated by mixtures containing copolymer components (even recycled ones), currently, their biggest disadvantage is the high production price. The results described in this work open up new paths through the use of waste PET, which until now seemed practically impossible. It is worth noting that the advantage of this solution is the reduction in financial outlays needed to purchase the polymer. By using an asphalt mixture containing MRPET in parts of streets, driveways, parking lots, and even sports fields, the city of Tarnów would obtain asphalt surfaces of excellent quality and save a significant part of the city budget by reducing the costs of road repair. For many years, great emphasis has been placed on recycling plastics. By using waste PET bottles to produce asphalt surfaces, the city of Tarnów would be the first city in Poland to use this method of processing plastics. Giving waste a second life is the best solution for a planet whose level of plastic pollution increases every year. The city of Tarnów could regain the reputation of being “environmentally friendly” by using modified recyclate (RPET) asphalt mixtures. Although the subject of polymer-modified asphalt mixtures still has a long way to go, from the idea through to laboratory tests to final implementation, it is already expected that the obtained polymer-modified asphalt mixtures will be characterized by greater resistance to cracking and rutting by increasing their flexibility at low temperatures and stiffness at high temperatures. The addition of waste PET can also extend the life cycle of an asphalt surface by increasing strength and resistance to abrasion, aging, and oxidation.

## Figures and Tables

**Table 1:** Properties and use of 50/70, 70/100 and 100/150 road bitumens.

Road Bitumens’ Type	Penetration at 25 °C (0.1 mm)	Softening Point (°C)	Application
50/70	50–70	46–54	A component of mastic–grit mixtures (SMA) in wearing courses
70/100	70–100	43–51	A component of mastic–grit mixtures (SMA) in wearing courses
100/150	100–150	39–47	Production of asphalt emulsions

**Table 2:** The values of elastic recovery (%) for bitumen types 50/70, 70/100, 100/150 heated to temperatures up to 150 °C and 240 °C.

Type of Road Construction Bitumen	Heating Temperature (°C)	d (mm)	R_E_ (%)
50/70	150	25	13
	240	10	5
70/100	150	35	18
	240	28	14
100/150	150	23	12
	240	33	17

**Table 3:** Samples obtained by adding MRPET and HCl to road bitumens.

Type of Road Construction Bitumen	Amount of Road-Construction Bitumen (wt%)	Amount of MRPET (wt%)	Amount of 10 M HCl (wt%)	Name of Sample
50/70	95.5	2	2.5	50/70/2RPET
	92.5	5		50/70/2RPET
	87.5	10		50/70/10RPET
70/100	95.5	2		70/100/2RPET
	92.5	5		70/100/5RPET
	87.5	10		70/100/10RPET
100/150	95.5	2		100/150/2RPET
	92.5	5		100/150/5RPET
	87.5	10		100/150/10RPET

**Table 4:** The values of elastic recovery (%) for bitumen types 50/70, 70/100, and 100/150 heated to temperatures up to 150 °C and 240 °C and successively with the addition of 2, 5, and 10 wt% MRPET.

Type of Road Construction Bitumen	Sample	d (mm)	R_E_ (%)
50/70	50/70	10	5
	50/70/2MRPET	21	11
	50/70/2MRPET	48	24
	50/70/10MRPET	29	15
70/100	70/100	28	14
	70/100/2MRPET	11	6
	70/100/5MRPET	31	16
	70/100/10MRPET	30	15
100/150	100/150	33	17
	100/150/2MRPET	30	15
	100/150/5MRPET	35	18
	100/150/10MRPET	35	18

**Table 5:** Microscopic photos (40× magnification) of samples: (A) 50/70, (B) 50/70/2MRPET, (C) 50/70/5MRPET, (D) 50/70/10MRPET.

(A) 50/70	(B) 50/70/2MRPET	(C) 50/70/5MRPET	(D) 50/70/10MRPET
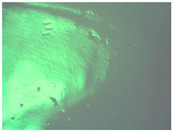	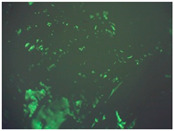	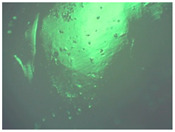	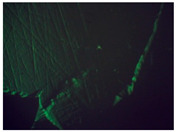

**Table 6:** Microscopic photos (40× magnification) of samples: (A) 70/100, (B) 70/100/2MRPET, (C)70/100/5MRPET, (D) 70/100/10MRPET.

(A) 70/100	(B) 70/100/2MRPET	(C) 70/100/5MRPET	(D) 70/100/10MRPET
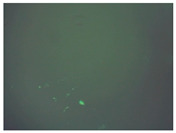	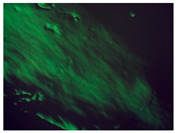	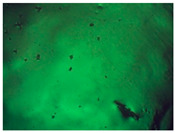	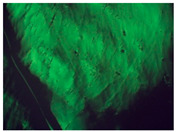

**Table 7:** Microscopic photos (40× magnification) of samples: (A) 100/150, (B)100/150/2MRPET, (C)100/150/5MRPET, (D) 100/150/10MRPET.

(A) 100/150	(B) 100/150/2MRPET	(C) 100/150/5MRPET	(D) 100/150/10MRPET
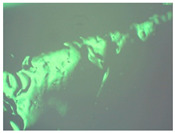	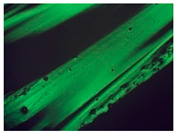	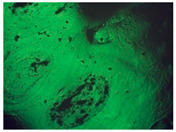	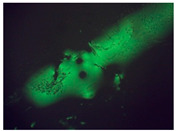

**Table 8:** The obtained samples of polymer-modified asphalt mixtures (PMA) with the addition of mineral filler.

Type of Road Construction Bitumen	Amount of Road Construction Bitumen (wt%)	Amount of MRPET (wt%)	Amount of Mineral Filler (wt%)		Amount of 10 M HCl (wt%)	Name of Sample
			MB	PB		
50/70	37.5	10	50	-	2.5	50/70/10MRPET/MB
			-	50		50/70/10MRPET/WB
70/100			50	-		70/100/10MRPET/PB
			-	50		70/100/10MRPET/WB
100/150			50	-		100/150/10MRPET/WB
			-	50		100/150/10MRPET/WB

**Table 9:** Names of samples of asphalt mixtures collected in the city of Tarnów.

Place of Taking a Sample of the Mineral-Modified Asphalt Mixtures	Name of Sample
Lwowska Street (50°00′52.5″ N 21°00′14.7″ E)	MMAL
Moscickiego Street (50°00′50.7″ N 20°57′41.5″ E)	MMAL
Kochanowskiego Street (50°00′21.5″ N 20°57′56.4″ E)	MMAL
Mrozna Street (50°02′30.5″ N 20°58′05.1″ E)	MMAMR

**Table 10:** Photos of samples before and after the resistance test to negative temperature.

Frost Resistance		
Sample	Before the Test	After the Test
50/70/10MRPET/PB	* 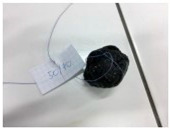 *	* 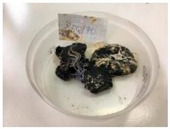 *
70/100/10MRPET/PB	* 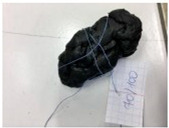 *	* 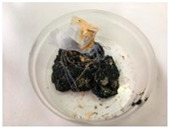 *
100/150/10MRPET/PB	* 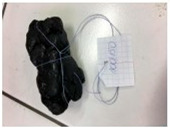 *	* 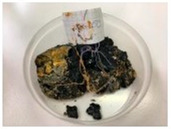 *
MMAL	* 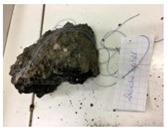 *	* 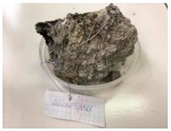 *
MMAM	* 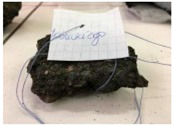 *	* 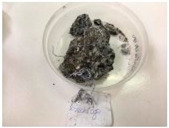 *
MMAK	* 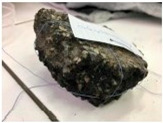 *	* 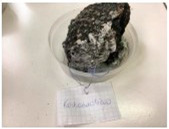 *
MMAMR	* 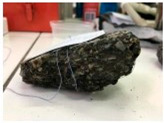 *	* 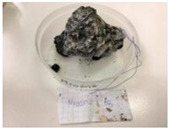 *

**Table 11:** Microscopic photos (40× magnification) before and after the temperature resistance test.

Structure		
Sample	Before the Test	After the Test
50/70/10MRPET/PB	* 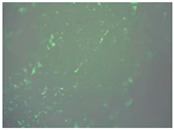 *	* 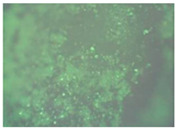 *
70/100/10MRPET/PB	* 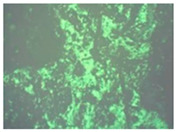 *	* 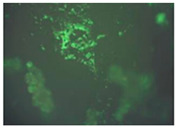 *
100/150/10MRPET/PB	* 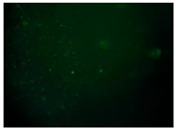 *	* 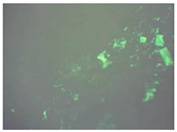 *
MMAL	* 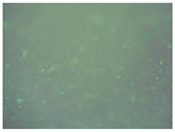 *	* 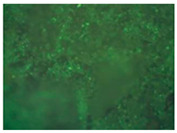 *
MMAM	* 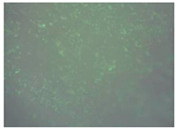 *	* 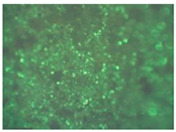 *
MMAK	* 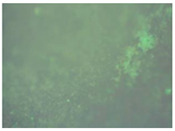 *	* 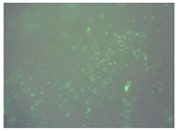 *
MMAMR	* 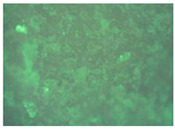 *	* 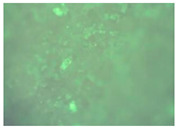 *

**Table 12:** Abrasion value (mm^3^/m) for mineral–polyester–asphalt mixtures and asphalt mixtures collected in the city of Tarnów.

Sample	Abrasion (X) (mm^3^/m)
50/70/10MRPET/PB	6.56
70/100/10MRPET/PB	1.02
100/150/10MRPET/PB	2.71
MMAL	1.89
MMAM	9.38
MMAK	6.07
MMAK	7.48

## Data Availability

The data are contained within the article.
